# SlWRKY16 and SlWRKY31 of tomato, negative regulators of plant defense, involved in susceptibility activation following root-knot nematode *Meloidogyne javanica* infection

**DOI:** 10.1038/s41598-023-40557-z

**Published:** 2023-09-05

**Authors:** Anil Kumar, Natalia Sichov, Patricia Bucki, Sigal Brown Miyara

**Affiliations:** Department of Entomology, Nematology and Chemistry Units, Agricultural Research Organization (ARO), Volcani Center, 50250 Bet Dagan, Israel

**Keywords:** Molecular biology, Plant sciences

## Abstract

The involvement of WRKY transcription factors in plant-nematode interactions, and in particular, how these WRKYs participate in regulating the complex morphological and physiological changes occurring after nematode infection, are the topic of active research. We characterized the functional role of the unstudied tomato WRKY genes *SlWRKY16* and *SlWRKY31* in regulating tomato roots’ response to infection by the root-knot nematode *Meloidogyne javanica*. Using promoter–GUS reporter gene fusions and qRT-PCR, we show that both *SlWRKY*s are predominantly expressed during the first half of the parasitic life stages, when feeding-site induction and construction occur. Expression of *SlWRKY16* increased sharply 15 days after inoculation, whereas *SlWRKY31* was already induced earlier, but reached its maximum expression at this time. Both genes were downregulated at the mature female stage. To determine biological function, we produced transgenic lines overexpressing *SlWRKY16* and *SlWRKY31* in tomato hairy roots. Overexpression of both genes resulted in enhanced *M. javanica* infection, reflected by increased galling occurrence and reproduction*.* Expression profiling of marker genes responsive to defense-associated phytohormones indicated reductions in salicylic acid defense-related *PR-1* and jasmonic acid defense-related *PI* in inoculated roots overexpressing *SlWRK16* and *SlWRKY31*, respectively. Our results suggest that *SlWRKY16* and *SlWRKY31* function as negative regulators of plant immunity induced upon nematode infection.

## Introduction

During the interaction between *Meloidogyne* spp. root-knot nematodes (RKNs) and their host, complex morphological and physiological changes occur in the infected plant tissue, ultimately resulting in the establishment of a nematode feeding site that supports nematode development^1^. Plants respond to root-knot nematode (RKN) infection by activating various immune responses that involve multiple recognition systems. One such system is the recognition of pathogen-associated molecular patterns (PAMPs) derived from plant-parasitic nematodes by cell surface-localized pattern-recognition receptors, leading to pattern-triggered immunity (PTI). Additionally, plants can detect tissue and cell damage caused by RKN invasion or migration through the recognition of damage-associated molecular patterns by pattern-recognition receptors^2^. Furthermore, plants possess resistance proteins that can directly or indirectly recognize pathogen effectors, triggering effector-triggered immunity (ETI)^3–5^. In both PTI and ETI, a crucial step is the extensive reprogramming of gene expression, which is regulated by various transcription factors (TFs) that play important roles in plant innate immunity^6^. Research findings have revealed that within plant genomes, there exists a substantial number of transcription factors (TFs) or transcriptional regulators, estimated to be around 1000–3000. These TFs make up a significant proportion, ranging from 5 to 15%, of all the encoded proteins in plants^7,8^. Transcriptomic studies have provided a comprehensive understanding of TF gene expression during plant defense against pathogens, shedding light on their roles in global gene-expression networks involved in plant defense mechanisms^9^. This knowledge about defense-related TFs contributes to our overall comprehension of plant defense responses to pathogen infections^10^.

The WRKY family of transcription factors is one of the largest in plants, playing crucial roles in plant growth, development, and response to environmental stresses^11^. WRKYs regulate disease-related processes through various mechanisms, such as enhancing physical barriers, modifying histones, regulating gene expression of pathogenesis-related genes, interacting with ROS signaling, and participating in crosstalk with phytohormones like salicylic acid (SA) and jasmonic acid (JA)^12^. These diverse functions enable WRKYs to control the initiation, progression, and severity of diseases either positively or negatively^12^.

The WRKY family of transcription factors is characterized by a highly conserved WRKY domain, which consists of the WRKYGQK motif at the N terminus and a zinc finger-like motif at the C terminus^13,14^. These WRKY proteins function as transcriptional activators or repressors in regulatory pathways by binding to the W-box *cis*-acting element (consensus sequence (T)(T)TGAC(C/T)) present in the promoter regions of their target genes^12^.

WRKYs are implicated in the two layers of induced defense responses^15^, where they act as either positive or negative regulators^12,16^. The first layer is known as PAMP-triggered immunity (PTI), which is initiated upon recognition of PAMPs by the plant’s pattern-recognition receptors and which might be subjected to suppression by the pathogen's effectors. The second layer is effector-triggered immunity (ETI), which is triggered when plant resistance (R) proteins recognize pathogen effectors, although it can also be countered by the pathogen's own effectors^14^. Both PTI and ETI induce local and systemic acquired resistance responses by generating reactive oxygen species (ROS) and activating a signaling network involving mitogen-activated protein kinases (MAPKs) and hormonal pathways^17^. The classical immunity-related hormones involved in this process are salicylic acid (SA), jasmonic acid (JA), and ethylene. Accumulating evidence demonstrates that WRKYs are involved in PTI and ETI at various regulatory levels^12^. Firstly, WRKYs can directly interact with PAMPs or effector proteins to activate or repress PTI and ETI responses^12^. Secondly, they can be regulated by MAPKs^18,19^. Thirdly, WRKYs play a role in modulating hormonal signaling^12,20^. Additionally, WRKYs contribute to plant immunity through the modulation of small RNAs, epigenetic mechanisms such as histone methylation, proteasome-mediated degradation, and inter-organelle retrograde signaling^12,20^.

In the genome of tomato (*Solanum lycopersicum*), a total of 83 SlWRKY genes have been identified^21,22^. Several WRKYs have been extensively studied to understand their involvement in plant defense through overexpression or silencing experiments^14^. Upon pathogen infection, altered expression patterns have been observed for various tomato WRKY genes, such as *SlWRKY23* (homologous to Arabidopsis *AtWRKY23*), *SlWRKY46* (homologous to *AtWRKY40*), *SlWRKY53*/*54* (homologous to *AtWRKY23*), *SlWRKY80*, and *SlWRKY81* (homologous to *AtWRKY38* and *AtWRKY62*, respectively)^21,23–25^. Arabidopsis homologs of these WRKYs have been analyzed, revealing their roles as negative regulators of plant defense. For example, *AtWRKY38*, *AtWRKY48*, and *AtWRKY62* are involved in the response to *Pseudomonas syringae*^26–28^, *AtWRKY23* in the response to the nematode *Heterodera schachtii*^1^, and *AtWRKY27* and *AtWRKY53* in the response to *Ralstonia solanacearum*^29,30^. Conversely, numerous WRKY genes in tomato have been identified as positive regulators of plant responses to biotic stresses. For instance, *SlWRKY31* (referred to as *SlDRW1* in Liu et al.^31^) and *SlWRKY33*^32^, which are homologs of *AtWRKY33*, restored the compromised tolerance of the atwrky33 mutant to *Botrytis cinerea*^33^. Furthermore, the overexpression of the *Solanum pimpinellifolium* allele of *SlWRKY33*^34^ conferred resistance to the hemi-biotrophic oomycetes *Phytophthora nicotianae* in tobacco and *Phytophthora infestans* in tomato. Another gene, *SlWRKY39*, which shares homology with *AtWRKY40*, showed significant upregulation in tomato plants when challenged with *P. syringae*^21^. Notably, tomato lines that overexpressed *SlWRKY39* exhibited enhanced resistance against this pathogen^35^.

Among the studied tomato WRKY genes, special attention has been paid to WRKYs implicated in regulating plant responses to the *Meloidogyne* sp. RKNs^14^. A previous comprehensive analysis of the transcriptional profile in tomato during its compatible response to the root-knot nematode *Meloidogyne javanica* revealed differential expression of WRKY genes^36^. Overexpression of *SlWRKY45,* which is a homolog of *AtWRKY40*, resulted in increased susceptibility of tomato plants to *M. javanica*. This susceptibility was accompanied by a decrease in the expression of marker genes associated with jasmonic acid (JA) and salicylic acid (SA) signaling pathways^37^. In contrast, *SlWRKY3*, which is a homolog of *AtWRKY4*, has been demonstrated to function as a positive regulator of resistance against *M. javanica*^38^. Additionally, *SlWRKY72*, *SlWRKY73*, and *SlWRKY74* have been shown to contribute positively to both PTI and *Mi-1*-mediated ETI against RKNs (*M. javanica*) and potato aphids (*Macrosiphum euphorbiae*)^39^. *SlWRKY80*, was found to be essential for *Mi-1-*mediated resistance against potato aphids and nematodes^40^. An intriguing question arises as to whether these WRKYs are involved in the instability of *Mi-1*-mediated resistance under heat stress or, more broadly, if WRKYs play a role in the (in) stability of plant resistance mediated by R genes associated with different molecular mechanisms^41^.

The precise roles of various WRKY TFs in tomato's response to RKNs and their interactions with multiple WRKY TFs in plant immunity are not fully understood. Therefore, further functional analysis of additional tomato *WRKY* genes is required to unravel the entire complex WRKY network—the black box that is engaged in promoting enhanced resistance or increased susceptibility to nematodes.

We carried out a functional study of *SlWRKY16*- and *SlWRKY31*-encoding genes, which belong to the WRKY-encoding group of 16 genes that was previously identified through a wide transcriptomic study and which demonstrated differential gene expression during *M. javanica* infection^37^. Whereas *SlWRKY16* has never been characterized, a previous study indicated that its expression is induced by SA^42^. *SlWRKY31* is a homolog of *AtWRKY48*, a stress- and pathogen-responsive transcriptional activator known to represses basal defense in *Arabidopsis* against the bacterial pathogen *P. syringae*^28^. To uncover these TFs’ involvement in regulating the host response to RKN infection, we studied the expression profile of both transcripts during the interaction of tomato roots with *M. javanica* and following wounding. Functional analysis through overexpression of *SlWRKY16* and *SlWRKY31* and their role in regulating defense-related hormone signaling suggest that both WRKYs are negative regulators of plant defense.

## Materials and methods

### Alignment of sequences and phylogenetic analysis

In the study conducted by Huang et al.^21^, the protein sequences of tomato SlWRKY16 (Solyc07g056280.2.1) and SlWRKY31 (Solyc05g053380.2.1) were classified as members of group IIc in the WRKY TFs, which consists of 16 members in tomato. The sequences of SlWRKY16 and SlWRKY31 were obtained from the Sol Genomics website (https://solgenomics.net/), and their active domains were subjected to analysis using the NCBI CDD server (http://www.ncbi.nlm.nih.gov/Structure/cdd/cdd). To determine sequence homology, the WRKY16 and WRKY31 sequences were employed in a comparative analysis across *Arabidopsis*, rice, and tomato. BLASTp and tBLASTn algorithms were executed, utilizing the Sol Genomics Network (https://solgenomics.net/), the Arabidopsis Information Resource TAIR (https://www.arabidopsis.org/), the Rice Gene Annotation Project (http://rice.plantbiology.msu.edu/), and the NCBI non-redundant protein sequence database (http://www.ncbi.nlm.nih.gov). The derived sequences of WRKY16 and WRKY31 were then exposed to simple phylogenetic analysis with MEGA (version 6) software using the neighbor-joining method^43^. Bootstrap values were determined through 1,000 repetitions to assess the phylogenetic tree's reliability. The WRKY accession numbers and tomato IDs of all WRKYs employed in the phylogenetic analyses are as follows: SlWRKY16 (Solyc07g056280.2.1), SlWRKY71 (Solyc02g071130.2), SlWRKY28 (Solyc12g011200.1), SlWRKY31 (Solyc05g053380.2.1), SlWRKY47 (Solyc01g058540.2.1), SlWRKY23 (Solyc01g079260.2.1); from *Arabidopsis thaliana*: AtWRKY71 (AT1G29860.1), AtWRKY28 (AT4G18170.1), AtWRKY57 (AT1G69310.1), AtWRKY48 (AT5G49520.1), AtWRKY8 (AT5G46350.1), AtWRKY23 (AT2G47260.1); from *Oryza sativa*: OsWRKY16 (LOC_Os01g47560.1), OsWRKY3 (LOC_Os03g55080.1), OsWRKY8 (LOC_Os05g50610.2), OsWRKY49 (LOC_Os05g49100.1).

### Regulatory element analysis of WRKY promoters

The upstream region of the SlWRKY genes, spanning 2.0 kb, was isolated from tomato genomic DNA obtained from the source (https://solgenomics.net/). To conduct in-silico analysis of the promoter sequences, the PlantCARE^44^ and PLACE^45^ databases were utilized as tools. These databases provided access to *cis-*acting DNA regulatory elements that bind to the promoter region of the investigated WRKY genes.

### Culturing of Nematodes and preparation of inoculum for infection assay

*Meloidogyne javanica* was cultured on tomato plants (*Solanum lycopersicum* cv. Avigail 870) in a greenhouse under controlled conditions. The plants were grown with a 16-h light regime at a temperature of 25 °C for a duration of 4 to 6 weeks. To extract the nematode eggs, a modified version of the method described by a previous study^46^ was employed. Tomato roots were thoroughly washed, cut into segments, and macerated in a blender with a 0.05% (v/v) sodium hypochlorite (NaOCl) solution for 3 min. The resulting suspension was then passed through a set of three sieves with mesh sizes of 120, 60, and 30 µm. After discarding the debris collected on the sieves, the eggs deposited on the 30-µm sieve were transferred to a 50-mL test tube. Centrifugal flotation, following the method of Hussey and Barker^47^, was performed. The supernatant containing the eggs was poured onto a 30-µm sieve, washed with tap water, and the eggs were collected into MES buffer. The collected eggs were then sterilized using the procedure outlined by van Vuuren and Woodward^48^. Subsequently, the sterilized eggs were transferred back onto a 30-µm sieve and enclosed in a petri dish with 5 mL of MES buffer. The petri dish was placed in a growth chamber at a 28 °C in complete darkness, allowing the eggs to hatch over a period of 5 to 6 days.

### Isolation of RNA and qRT-PCR analysis

Tomato roots were collected at various time points (0, 1, 2, 3, 10, 15, and 28 days post-infection) after *M. javanica* infection to isolate RNA. The RNA extraction was performed using Invitrogen TRIzol reagent (Thermo Fisher Scientific, Carlsbad, CA, US). To eliminate potential genomic DNA contamination, the RNA samples were subjected to TURBO DNA-free DNase treatment (Applied Biosystems, Foster City, CA) following the manufacturer's instructions. The quantification of gene transcripts for *WRKY16* and *WRKY31* was carried out using real-time reverse transcription PCR (qRT-PCR) on the total RNA obtained at the mentioned time points. Initially, the synthesis of the cDNA strand was performed using 1 µg of total RNA and the SuperScript III cDNA kit (Thermo Fisher Scientific). The qRT-PCR was carried out with SYBR-Green ROX Mix (ABgene, Epsom, UK), and primers for gene quantification were designed using primer3plus (https://bioinfo.ut.ee/primer3-0.4.0/). The qRT-PCR reaction contained 2 µL of cDNA in a total volume of 10 µL was performed as per Chinnapandi et al.^38^, and the PCR cycles consisted of an initial step of 2 min at 50 °C, followed by 10 min at 95 °C, and then 40 two-step cycles of 10 s at 95 °C and 1 min at 60 °C. For the qRT-PCR, a mixture of all cDNAs was used as a template for calibration curves specific to each primer pair. Each reaction was conducted in triplicate, and the results represent the average of three independent biological experiments. The expression levels of two constitutively expressed genes, *β-tubulin* (GenBank accession no. NM_001247878.1), and *β-actin* (GenBank accession no. U60482.1) were used as endogenous controls for gene-expression analyses in tomato. The transcript levels of each sample were standardized by using the geometric mean of specific housekeeping genes^38^. To validate the qRT-PCR results, the expression of a subset of genes was assessed in two additional independent experiments, yielding consistent outcomes. The expression levels for each treatment were determined relative to the control noninoculated roots.

### Plasmid construction and generation of transgenic hairy roots

For the promoter–GUS reporter assays, the promoter regions (about 1.5 kb upstream of the ATG) of *SlWRKY16* (1436 bp) and *SlWRKY31* (1496 bp) were amplified from *S. lycopersicum* genomic DNA using the gene-specific primers (Table S1). After amplification, the genomic fragments obtained were cloned into the pGEMT vector (Promega) and validated through sequencing. Subsequently, the promoter fragment was amplified from the pGEMT vector in both orientations using primers containing appropriate attB sites. The amplified fragment was then cloned into the pDONR221 vector through recombination using a Gateway BP kit (Invitrogen). Finally, the promoter fragment was transferred into the pKGWFS7^49^ destination vector through recombination using a Gateway LR kit, resulting in the generation of transcriptional fusions that drive GFP-GUS expression.

To facilitate the overexpression of *SlWRKY16* and *SlWRKY31*, the coding sequences were amplified from tomato plant cDNA derived from isolated total RNA. Gene-specific primers containing attB sites (Supplementary Table S1) were utilized to flank the coding sequences. The resulting PCR products, encompassing the full-length *SlWRKY16* (968 bp) and *SlWRKY31* (888 bp) sequences, were then inserted into the pDONR221 vector using BP clonase (Invitrogen, Carlsbad, CA). Subsequently, the pDONR221 vector harboring both WRKY genes underwent recombination with the pK7WG2D1^49^, vector using LR clonase (Invitrogen). The resulting constructs were transformed into *Escherichia coli* (DH5α) cells, and individual colonies were subjected to PCR screening to confirm the presence of inserts. Selected clones were further verified by sequencing analysis. These constructed vectors, including an empty vector control, were subsequently employed for *Rhizobium rhizogenes*-mediated root transformation, following the provided protocol. All primers utilized in this study were synthesized by integrated DNA technologies.

### The transformation of roots using* R. rhizogenes* and the generation of hairy root cultures

The binary vectors for promoter analysis, pKGWFS7::WRKY16:GUS and pKGWFS7::WRKY31:GUS, together with pK7WG2D,1::OEWKRY16, pK7WG2D,1::OEWKRY31 and empty vector controls (pKGWFS7 and pK7WG2D,1) were introduced into *R. rhizogenes* ATCC 15834 through electrotransformation^50^. Cotyledons were individually extracted from tomato seedlings aged 15 to 20 days and submerged in a suspension of *R. rhizogenes* that had been incubated for 2 days at 28 °C, with agitation at 100 rpm. The excised cotyledons were then placed on standard-strength Gamborg's B5 salts medium for a co-cultivation period of 3 days. Subsequently, they were transferred to B5 agar media supplemented with kanamycin at 50 mg/mL (Duchefa, Haarlem, the Netherlands) and timentin (15:1) at 300 mg/mL (Duchefa). Following an incubation period of 7–10 days in the dark at 25 °C, roots began to emerge from the wounded surface of the cotyledons, indicating successful transformation. The hairy roots obtained were subsequently transferred to Gamborg's B5 medium (GB) supplemented with 0.8% (w/v) Gelrite and kanamycin at 50 mg/mL. To confirm the presence of transgenic lines, DNA and RNA from the respective constructs were analyzed using vector-specific GUS and GFP primers (Supplementary Table S1). In the nematode-infection experiments, the transformed roots were subcultured in media without antibiotics for a duration of 2 weeks. Following this, 200 freshly hatched sterile *M. javanica* juveniles were introduced to inoculate the transgenic root lines. At specified time intervals, root samples were collected for GUS assessment and disease evaluation. The presence and expression of the transgenes in the tomato hairy roots overexpressing *WRKY16* and *WRKY31* were verified using qRT-PCR analysis, employing the primers provided in Table S2.

### Localization of GUS activity through histochemical staining and microscopic examination

The 15-days-old transformed promoter-GUS root lines were subjected to *M. javanica* infection using the provided protocol. GUS activity was evaluated at 2, 5, 10, 15, and 28 days post-infection (dpi), with non-infected plants serving as controls in the promoter-GUS experiment. At the designated time points, infected and non-infected transgenic roots were collected from Gamborg's media and incubated with GUS buffer (composed of 50 mM sodium phosphate pH 7.0, 10 mM EDTA, 5 mM K_4_[Fe_2_(CN)_6_], 5 mM K_3_[Fe_2_(CN)_6_], 0.2% v/v Triton X-100, and 2 mM 5-bromo-4-chloro-3-indolyl β-d-glucuronide) for 12 h at 37 ºC^51,52^. Afterward, the roots were washed twice with distilled water. GUS expression was assessed in a minimum of 15–20 infected transgenic roots at each time point. To examine the cellular localization of GUS expression in giant cells, thin sections were prepared from infected roots at 15 and 28 dpi and examined under a microscope following the established procedure described in reference^53^.

To prepare the GUS-stained roots for histological sectioning, they were first dehydrated in a solution containing 0.25% (w/v) glutaraldehyde and 4% (v/v) paraformaldehyde in 50 mM PBS at pH 7.2. Subsequently, the dehydrated roots were embedded in Technovit 7100 according to the manufacturer's instructions, following the protocol described by Chinnapandi et al.^38^. For capturing GUS-stained root samples at 15 and 28 dpi, a stereomicroscope (Leica MZFLIII, Leica Microsystems GmbH) equipped with a Nikon DS-Fi1 camera was utilized. To conduct the wounding treatment, transgenic roots were initially subcultured in GB media for one week. Using sterile forceps, mechanical wounds were created at various locations along the length of the roots. The wounded roots were then returned to the GB plates and kept in a dark environment. At specific time intervals of 0, 9, and 24 h after GUS staining, the wounded roots were collected and photographed for further analysis.

### Evaluation of tomato overexpression roots in response to nematode infection

To assess the nematode infection in both control and WRKY-overexpressing transgenic lines, root samples were collected from the monoxenic culture after 28 days post-infection (dpi). The degree of nematode infection was determined by counting the number of galls and eggs per gram of root weight, following the methodology described in previous studies^54–57^. The average values of galls and eggs were obtained by analyzing 10–15 replicates per line. The root-infection experiments were replicated twice, and the results of one experiment are presented here. The data were statistically analyzed for significance, and differences among the means were determined using the Tukey–Kramer test with an alpha level of 0.05, using JMP software (SAS Institute, Cary, NC). To investigate the expression of defense genes in the overexpressing lines after infection, RNA was extracted from the roots 24 h post-infection with *M. javanica*, following the previously described protocol.

### Genomic DNA and cDNA isolation

For the amplification of the promoter regions of WRKY16 and WRKY31, genomic DNA was isolated from 1-month-old soil-grown tomato cv. Avigail 870 seedlings using the cetyltrimethylammonium bromide method, following the procedure described by Goetz et al.^58^. To generate full-length cDNA of *SlWRKY16* and *SlWRKY31*, tomato roots were finely ground in liquid nitrogen, and total RNA was extracted using Invitrogen TRIzol reagent (Thermo Fisher Scientific, Carlsbad, CA). To eliminate potential genomic DNA contamination, the RNA samples were subjected to TURBO DNA-free DNase treatment (Applied Biosystems, Foster City, CA) following the manufacturer's instructions. Subsequently, the DNA-free RNA was reverse transcribed into first-strand cDNA using the Verso cDNA kit.

### Statement of compliance

All experimental research on plants and nematodes described here complies with relevant institutional, national, and international guidelines and legislation. Formal ethical approval is not required.

## Results

### In-silico identification and characterization of SlWRKY16 and SlWRKY31

To gain a deeper understanding of the involvement of WRKY TFs in the process of RKN parasitism in tomato, we conducted a study to explore the functional characteristics of two WRKY genes that showed differential expression in tomato roots following *M. javanica* infection, as revealed by a previous RNA-Seq analysis^37,46^. These two WRKY genes are *SlWRKY16* and *SlWRKY31*. In-silico analysis revealed that SlWRKY16 is composed of 323 amino acids spanning three exons, featuring a single WRKY domain (WRKYGQK) and a zinc finger-like motif ligand, C-X4-C-X23-H-X1-H. Similarly, SlWRKY31 consists of 295 amino acids distributed across three exons, with a single WRKY domain (WRKYGQK) and a zinc finger-like motif ligand C-X4-C-X23-H-X1-H. Both proteins carry a nuclear localization signal, RKYGQK, at positions 190 and 160 of SlWRKY16 and SlWRKY31, respectively. According to Huang et al.^21^, both SlWRKY16 and SlWRKY31 were classified to WRKY group IIc.

**Figure 1:**
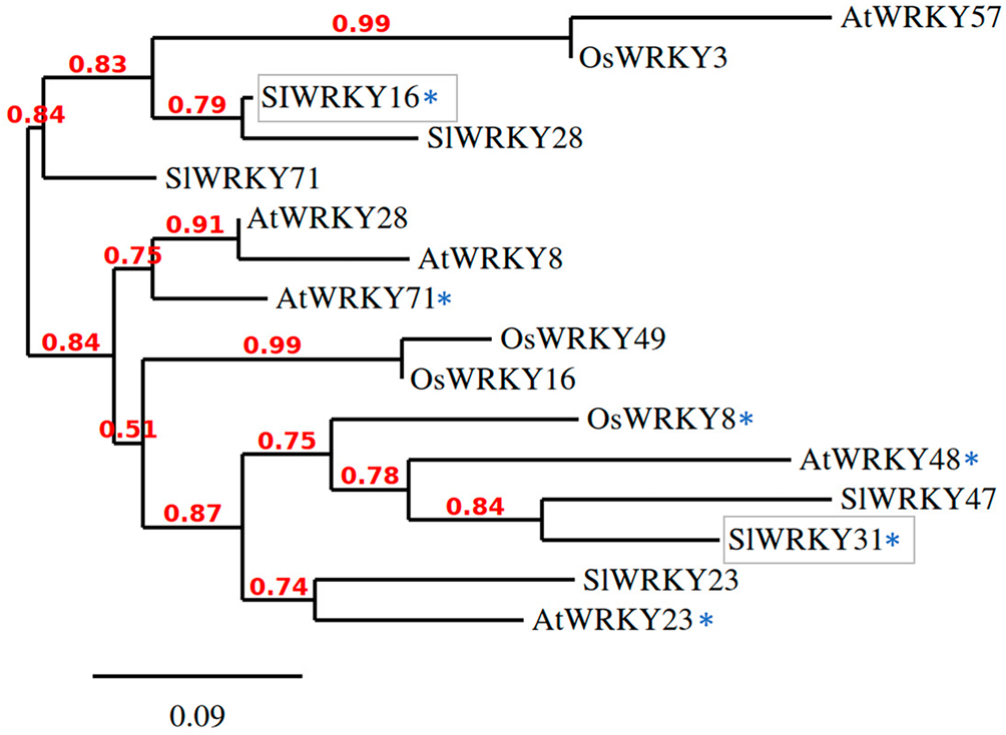
Phylogenetic tree of SlWRKY16 and SlWRKY31 generated using the neighbor-joining method with bootstrap value of 1,000 in MEGA (version 6) software. Phylogenetic analysis was carried out on WRKY genes from *Arabidopsis* (*AtWRKY*), rice (*Oryza sativa-OsWRKY*) and tomato (*SlWRKY*). Phylogenetic tree representing homology among *WRKY* genes from tomato, *Arabidopsis* and rice. *Negative regulator of plant immunity.

Phylogenetic analysis of both studied SlWRKYs was conducted by neighbor-joining method using a bootstrap value of 1000. A genome-wide NCBI-BLAST search of WRKY16 and WRKY31 revealed their homologs in tomato, rice and *Arabidopsis*. WRKY16 shared the highest sequence homology with three other WRKY proteins of tomato—SlWRKY71, SlWRKY31 and SlWRKY28, with 49.14, 42.15 and 58.75% similarity, respectively. Similarly, WRKY31 had the highest sequence homology with three other tomato WRKY proteins—SlWRKY23, SlWRKY47 and SlWRKY16, with 36.64, 51.13 and 42.15% similarity, respectively. Orthologs of SlWRKY16 were reported in rice (OsWRKY3, WRKY8 and WRKY16) and *Arabidopsis* (AtWRKY57, WRKY23, WRKY48, WRKY28, WRKY8 and WRKY71; Fig. 1); similarly, SlWRKY31 had several orthologs in rice (OsWRKY49, WRKY16, WRKY8 and WRKY3) and *Arabidopsis* (AtWRKY28, WRKY71, WRKY48 and WRKY23) (Fig. 1). Interestingly, SlWRKY16 and SlWRKY31 exhibited significant homology (40.62% and 43.06%, respectively) with the well-characterized AtWRKY48, a negative regulator of plant immunity against the bacterial pathogen *Pseudomonas syringae*.

### In-silico promoter analysis of SlWRKY16 and SlWRKY31

A 1436-bp and 1496-bp 5′-flanking sequence upstream of the *SlWRKY16* and* SlWRKY31* translation site (ATG), respectively, were analyzed using the PlantCARE and PLACE databases to investigate the regulatory elements and potential core sequences of the studied promoters (Table 1). The analysis revealed the presence of two primary types of *cis-*acting elements, specifically stress-responsive and hormone-responsive regulatory elements, within both promoters (as indicated in Table 1). Notably, several putative *cis*-acting elements associated with stress responses were identified, including the TATC-box, TCA element, TGA element, and the pathogenesis-related element BIHD1OS. Additionally, various motifs related to hormone responsiveness were discovered, such as the gibberellin-responsive elements TATC-box, WRKY71OS, and PYRIMIDINEBOXOSRAMY1A; the SA-responsive elements TCA element and WBOXATNPR1; the auxin-responsive TGA element; the ethylene-responsive element ERE; and the abscisic acid-responsive element ABRE. All *cis*-elements, along with their respective numbers and genomic locations, are listed in Table 1.

**Table 1:** Identification of hormone-specific *cis*-regulatory elements in *WRKY16* and *WRKY31* promoter regions using PLACE and PlantCARE databases.

*Cis*-element	Description	Sequences	Gene	
			*SlWRKY16*	*SlWRKY31*
TATC-box	Gibberellin-responsive element	TATCCCA and AGACAAA	1	1
WRKY71OS	Transcriptional repressor of the gibberellin signaling pathway	TGAC	3	9
Pyrimidineboxosramy1A	Gibberellin-responsive element 1	CCTTTT	3	2
TCA element	*Cis*-acting element involved in salicylic acid responsiveness	CCATCTTTTT	1	1
WBOXATNPR1	W-box recognized specifically by salicylic acid-induced WRKY DNA-binding protein	TTGACC	1	1
TGA element	Auxin-responsive element (AuxRE)	TGTCTC	1	1
ERE	Ethylene-responsive element	ATTTTAAA	1	2
ABRE	*Cis-*acting element involved in abscisic acid responsiveness	ACGTG	1	3
BIHD1OS	Pathogenesis-related	TGTCA	2	6

Element repeats are noted according to their occurrence.

### Spatiotemporal expression pattern of *SlWRKY16* and *SlWRKY31* during *M. javanica* root infection

**Figure 2:**
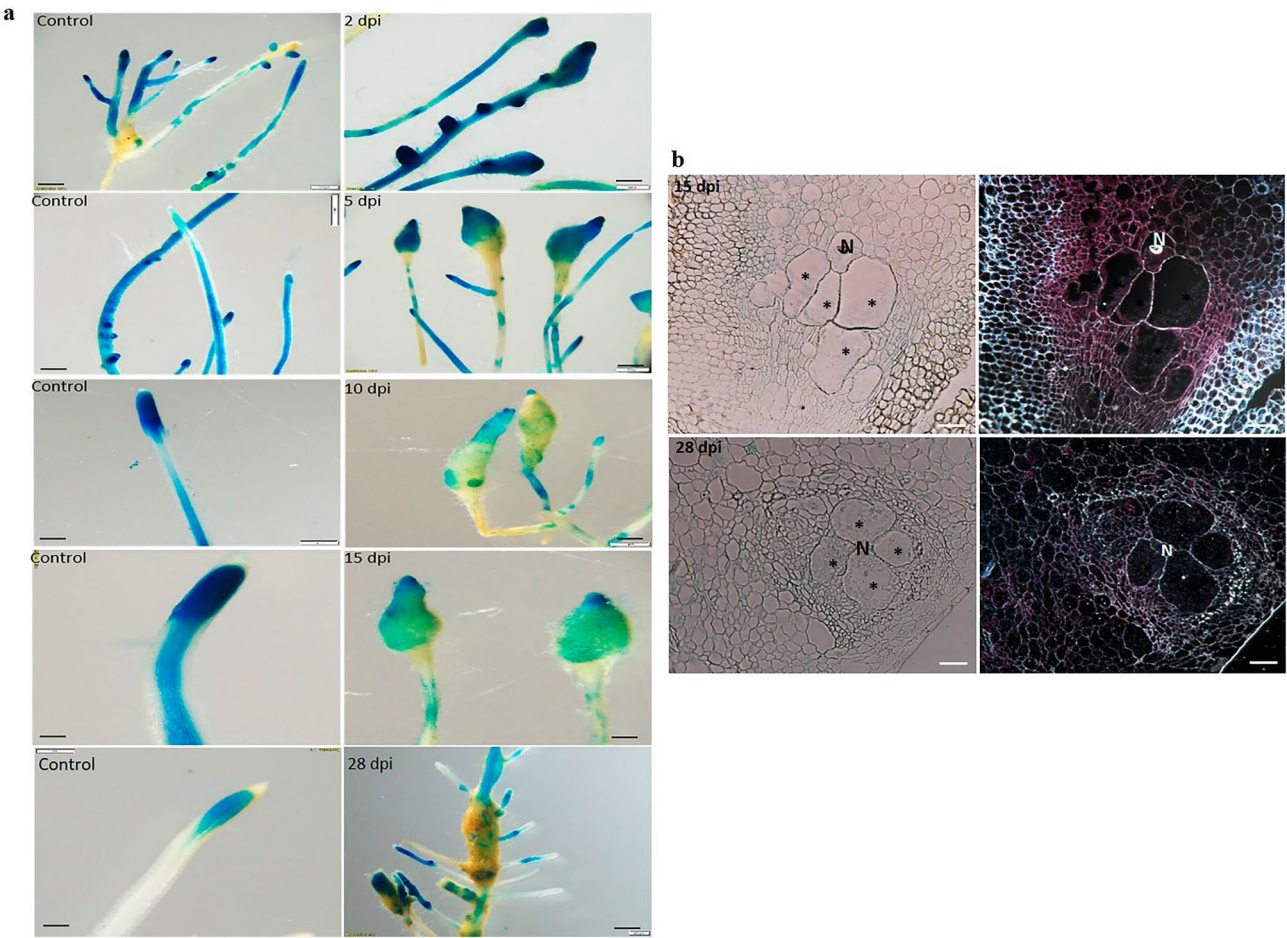
(**a**) GUS expression of *SlWRKY16* after *M. javanica* infection. GUS staining of promoter::GUS lines for WRKY16 was performed 2, 5, 10, 15 and 28 dpi and in uninfected roots (control), bar = 1 mm. (**b**) Cross-sections of *M. javanica*-infected root showing *SlWRKY16* expression 14 and 28 dpi. N, nematode juveniles; *, giant cell. Bar = 20 μm.

The full-length promoter fragments were obtained from tomato DNA and utilized in subsequent cloning procedures, resulting in the generation of binary vectors containing a promoter–GUS fusion construct. These vectors were then employed for the production of WRKY16::GUS and WRKY31::GUS tomato hairy root reporter gene lines through *Rhizobium rhizogenes*-mediated transformation, as described in detail in the Materials and Methods section. In order to examine the expression patterns of SlWRKY genes following inoculation, GUS signal in the root tissues was assessed at 2, 5, 10, 15, and 28 days post-inoculation (dpi) with second-stage juveniles (J2). The observed GUS expression in the inoculated root lines was compared with that in the respective noninoculated control root lines (Fig. 2a). WRKY16::GUS lines induced clear GUS expression (as observed by the blue signal at 2–15 dpi), associated with penetration and migration in the root-elongation zones in which tissue swelling occurred. At a later time point during nematode maturation and feeding-site establishment, GUS signal associated with the generated gall decreased significantly (28 dpi). GUS signal was primarily detected in the root-elongation zone and along the vasculature of noninoculated control roots (Fig. 2a). To investigate the role of SlWRKY16 in feeding-site establishment, thin sections of galls expressing SlWRKY16 promoter–GUS constructs were examined at 15 and 28 dpi. At 15 dpi, SlWRKY16 exhibited expression in the endodermis, pericycle cells, and phloem, with a noticeable signal in the developing feeding sites, as indicated by the red precipitate observed under dark-field optics (Fig. 2b). At 28 dpi, the expression level became significantly weaker in the pericycle cells and phloem, as evidenced by the GUS signal observed under light- and dark-field microscopy (Fig. 2b). These findings were consistent with the observations made on the entire root samples.

**Figure 3:**
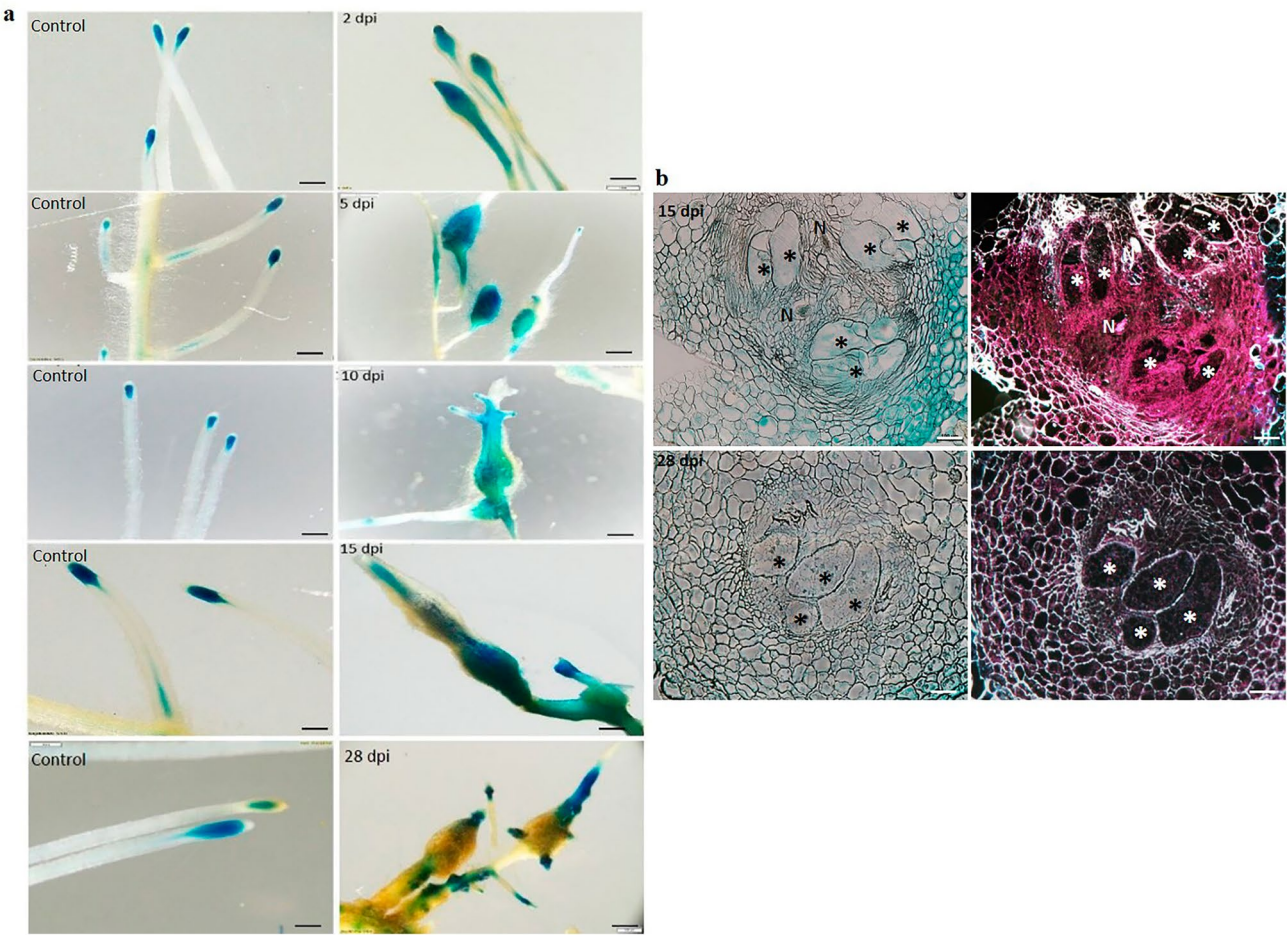
(**a**) GUS expression of *SlWRKY31* after *M. javanica* infection. GUS staining of promoter::GUS lines for WRKY31 was performed 2, 5, 10, 15, and 28 dpi and in uninfected roots (control), bar = 1 mm. (**b**) Cross-sections of *M. javanica*-infected root showing *SlWRKY31* expression 15 and 28 dpi. N, nematode juveniles; *, giant cell. Bar = 20 μm.

GUS analysis of WRKY31::GUS lines at 2 dpi showed increased expression in the root-elongation zone at the site of penetration and nematode migration (Fig. 3a). At 5 dpi, a pronounced GUS signal was detected in both the vascular system and the swollen root tissue, indicating the presence of nematode invasion (Fig. 3a); this signal remained very strong until 15 dpi. At 28 dpi, GUS signal in all infected root parts gradually diminished, with only residual GUS signal remaining in the developed galls (Fig. 3a). The control roots showed GUS expression in the apical meristem and the elongation zone at all-time points (Fig. 3a).

Thin sections of the SlWRKY31::GUS gall clearly showed high expression of *WRKY31* at 15 dpi. The expression was particularly prominent in the cortex and pericycle cells adjacent to the protoxylem poles, encompassing the developing giant cells. This expression pattern was clearly visualized through light- and dark-field microscopy, as evident from the intense GUS signal (Fig. 3b). Notably, the giant cells within the feeding sites associated with the developing nematodes at 15 dpi also displayed a strong GUS signal (Fig. 3b). At 28 dpi, the expression levels decreased such that only a weak signal remained.

In summary, it appears that *SlWRKY16* and *SlWRKY31* GUS expression peaked at 15 dpi and then declined, indicating that these genes’ expression underlies the changes following RKN infection.

### Expression of *SlWRKY16* and *SlWRKY31* is associated with the first half of the parasitic stage

**Figure 4:**
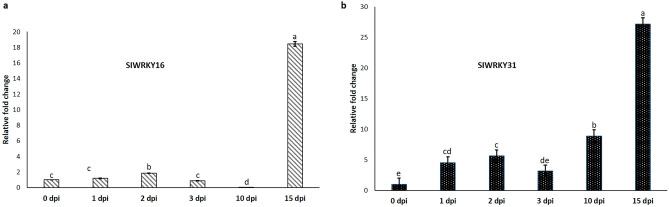
Expression of tomato WRKY genes in response to *M. javanica* infection. Expression of *SlWRKY16* (**a**) and *SlWRKY31* (**b**) genes in tomato roots was determined by qRT-PCR at 1, 2, 3, 10 and 15 dpi and in uninfected control root segments (0 dpi). Data include three independent biological and three technical replicates. *WRKY16* and *WRKY31* expression values are relative to uninfected control roots and were normalized using tomato *β-tubulin* as a reference gene. Bars indicate SEM. Different letters above the bars indicate statistically significant differences based on Tukey's HSD test (P < 0.05).

To determine when, after inoculation, *WRKY16* and *WRKY31* exhibit their main role, the expression pattern of both genes was evaluated using qRT-PCR in noninoculated control tomato roots and those 1, 2, 3, 10, and 15 dpi. *SlWRKY16* transcript was dramatically induced at 15 dpi (in the presence of third- and fourth-stage juveniles), increasing 18.44-fold compared to noninoculated roots and earlier time points post-infection (Fig. 4a). Expression of *SlWRKY31* increased gradually after inoculation, peaking at 15 dpi (27.1-fold change) (Fig. 4b). Expression of both genes declined at 28 dpi (Supplementary Fig. S1). These results further support the promoter–GUS assay, indicating that *WRKY16* and *WRKY31* are involved in plant processes that occur mainly at the infection stage when mature feeding sites have been constructed**.**

### Wound-response cues differentially regulate *SlWRKY16* and *SlWRKY31* expression

**Figure 5:**
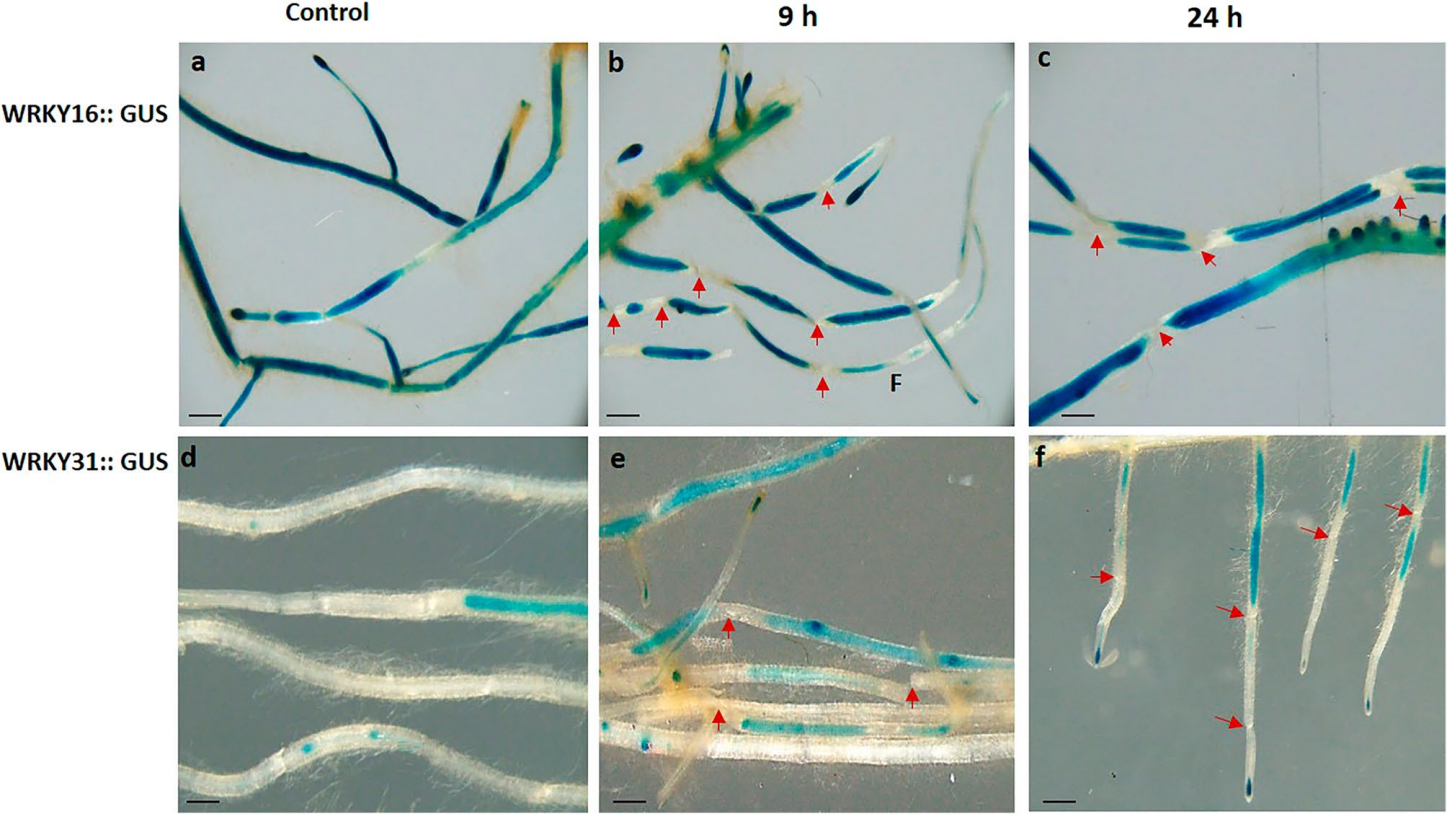
Expression patterns of *WRKY16* and *WRKY31* in transgenic lines under basal and wound-induced conditions. Histochemical GUS staining of WRKY16::GUS in control unwounded roots (**a**) and in roots 9 h (**b**) and 24 h (**c**) after mechanical wounding. Histochemical GUS staining of WRKY31::GUS in unwounded control roots (**d**) and in roots 9 h (**e**) and 24 h (**f**) after mechanical wounding. Scale bar = 1 mm. Red arrows indicate the location of mechanical wounding.

To explore the possible regulation of SlWRKY16 and SlWRKY31 promoter by wounding, we performed expression analyses using reporter gene GUS constructs fused to the promoters of *SlWRKY16* and *SlWRKY31* genes. Analysis of WRKY16 in nonwounded control roots showed uniform and constant GUS expression throughout the branching roots (Fig. 5a), whereas no activity of the WRKY16 promoter was detected at the site of wounding, both at 9 h (Fig. 5b) and 24 h (Fig. 5c) post-wounding. These findings imply that the expression of WRKY16 is specifically suppressed at the site of mechanical damage, indicating a localized response to the wound. In contrast, the promoter of WRKY31 did not show a significant increase in GUS activity at 9 h or 24 h after wounding compared to the unwounded control root (Fig. 5d–f).

### Overexpression of *SlWRKY16* and *SlWRKY31* promotes susceptibility to the RKN *M. javanica* in tomato

**Figure 6:**
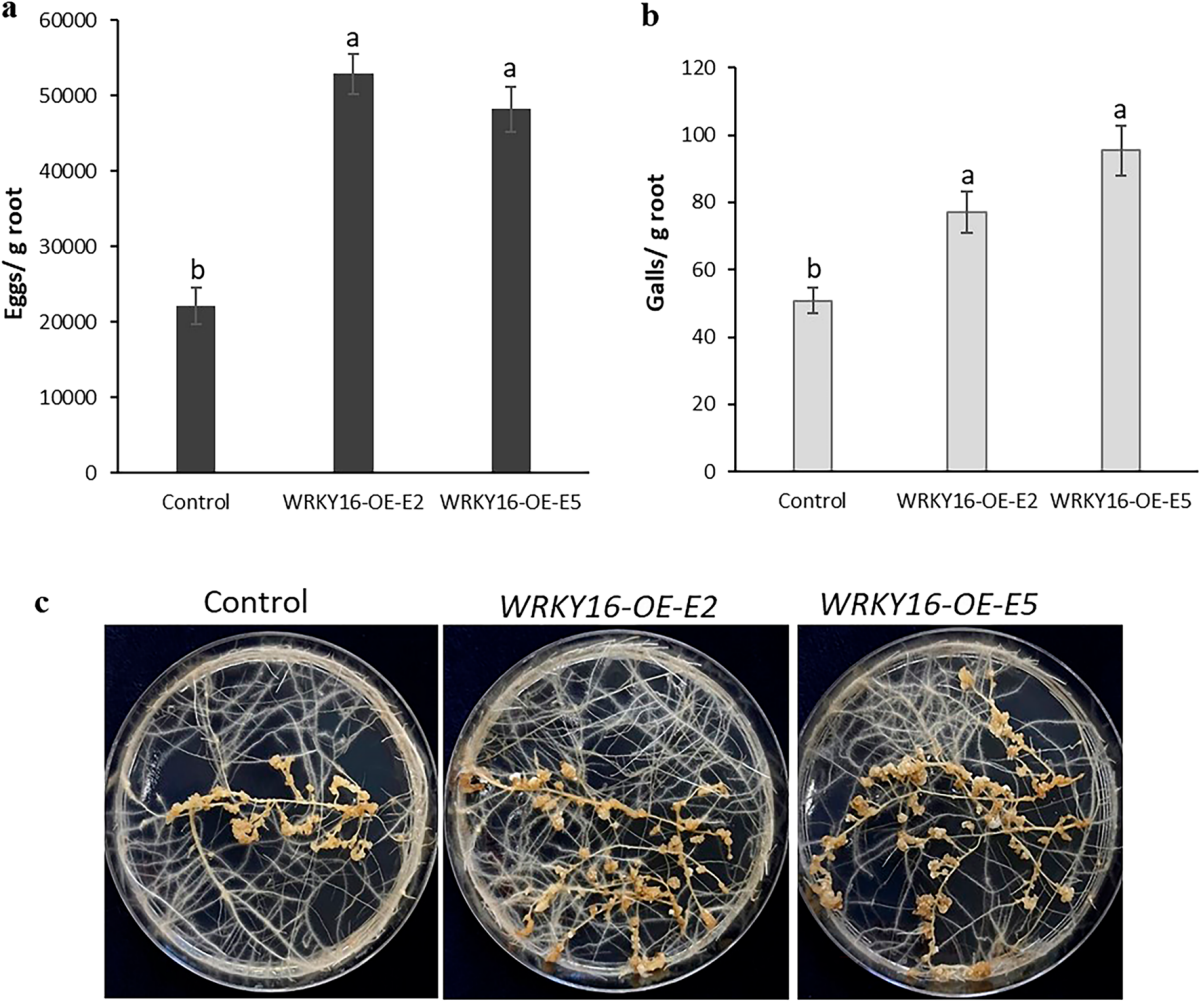
*SlWRKY16* overexpression in tomato hairy roots enhances *M. javanica* nematode infestation. Increased susceptibility of tomato hairy roots overexpressing *SlWRKY16* (WRKY16-OE-E2 and WRKY16-OE-E5) expressed as increased infection reflected by number of (**a**) eggs and (**b**) galls compared to the control line (empty vector). (**c**) Susceptibility of tomato roots overexpressing *SlWRKY16* to nematodes compared to control line. All root lines were subjected to inoculation with 500 sterile preparasitic J2s, and the infected roots were examined 28 days post-infection (dpi) to evaluate gall and egg development using a dissecting microscope. It is worth noting the significant (P < 0.05) increase in the percentage of eggs and galls in the WRKY16-OE-E2 and WRKY16-OE-E5 root lines compared to the control group. The data are presented as the means of 15 plants from each line, and the experiment was repeated three times, yielding consistent outcomes. The SEM represents the percentage of each developmental stage. Different letters above the bars indicate significant differences (P < 0.05, ANOVA) among the hairy root lines, as determined by Tukey–Kramer multiple comparison tests.

**Figure 7:**
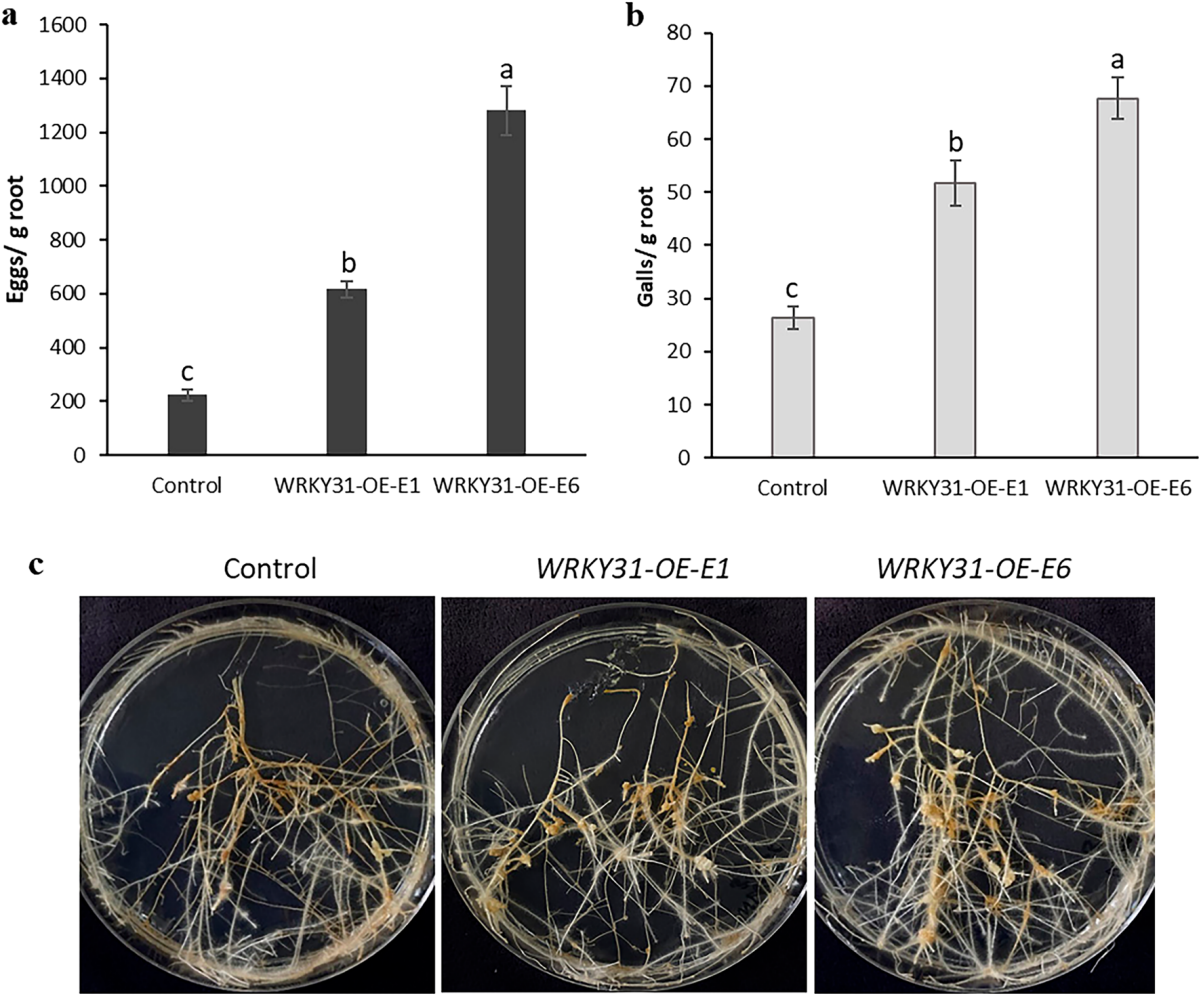
*SlWRKY31* overexpression in tomato hairy roots enhances *M. javanica* nematode infestation. Increased susceptibility of tomato hairy roots overexpressing *SlWRKY31* (WRKY31-OE-E1 and WRKY31-OE-E6) expressed as increased infection reflected by number of (**a**) eggs and (**b**) galls compared to the control line (empty vector). (**c**) Susceptibility of tomato roots overexpressing *SlWRKY31* compared to control line. All root lines were subjected to inoculation with 200 sterile preparasitic J2s, and the infected roots were examined 28 days post-infection (dpi) to evaluate gall and egg development using a dissecting microscope. It is important to note the significant (P < 0.05) increase in the percentage of eggs and galls observed in the WRKY31-OE-E1 and WRKY31-OE-E6 root lines compared to the control roots. The data are presented as the means of 15 plants from each line, and the experiment was repeated three times, yielding consistent results. The SEM represents the percentage of each developmental stage. Different letters above the bars indicate significant differences (P < 0.05) among the hairy root lines as determined by Tukey–Kramer multiple comparison tests.

To investigate the potential involvement of *WRKY16* and *WRKY31* in regulating plant responses to nematode infection, tomato plants were transformed using *R. rhizogenes*-mediated transformation with a plant binary vector containing a 969-bp and 888-bp fragment of *SlWRKY16* and *SlWRKY31*, respectively, under the control of the CaMV35S promoter. A total of 15 putative transformants resistant to kanamycin were obtained and maintained on selective media. Among the kanamycin-resistant transgenic lines carrying the 35S:SlWRKY16 construct, two lines (WRKY16-OE-E2 and WRKY16-OE-E5) with high transcript levels, as confirmed by qRT-PCR analysis (Fig. S2a), were selected for further investigation. Similarly, two lines (WRKY31-OE-E1 and WRKY31-OE-E6) overexpressing WRKY31, showing significant transcript levels (Fig. S2b), were also chosen for subsequent analysis. Notably, the transgenic roots did not exhibit any visible phenotypic differences compared to the control roots (Fig. S3a and b). These selected lines were then subjected nematode-inoculation assays to determine their degree of susceptibility to *M. javanica*. A 3-week-old root culture was inoculated with infective J2 nematodes, and the number of galls and eggs per gram root was determined 28 dpi and used to evaluate the involvement of *WRKY16 *and *WRKY31* in regulating plant susceptibility. Notably, the *SlWRKY16*-overexpressing lines were hyper-susceptible to *M. javanica* as indicated by increased reproduction on the respective roots as measured by egg counting, resulting in a 96 to 113% for WRKY16::*OE-E2* and WRKY16::*OE-E5,* respectively compared to control (empty vector) roots (Fig. 6a,c). Similarly, gall numbers increased by 41 to 69% for WRKY16-*OE-E2* and WRKY16-*OE-E5,* respectively compared to control roots (Fig. 6b,c). *SlWRKY31*-overexpressing lines also showed hyper-susceptibility to *M. javanica* as indicated by an increase in reproduction by143 to 385% for WRKY31-*OE-E1* and WRKY31-*OE-E6*, respectively compared to control (empty vector) roots (Fig. 7a,c). Similarly, gall counts showed an 80 to 131% increment for WRKY31-*OE-E1* and WRKY31-*OE-E6*, respectively compared to control roots (Fig. 7b,c). This hyper-susceptible phenotype suggests that increased expression of *WRKY16* and *WRKY31* plays an important role in mediating plant susceptibility to *M. javanica* infection.

### *SlWRKY16-* and *SlWRKY31*-overexpressing root lines show alterations in hormone-responsive gene expression

**Figure 8:**
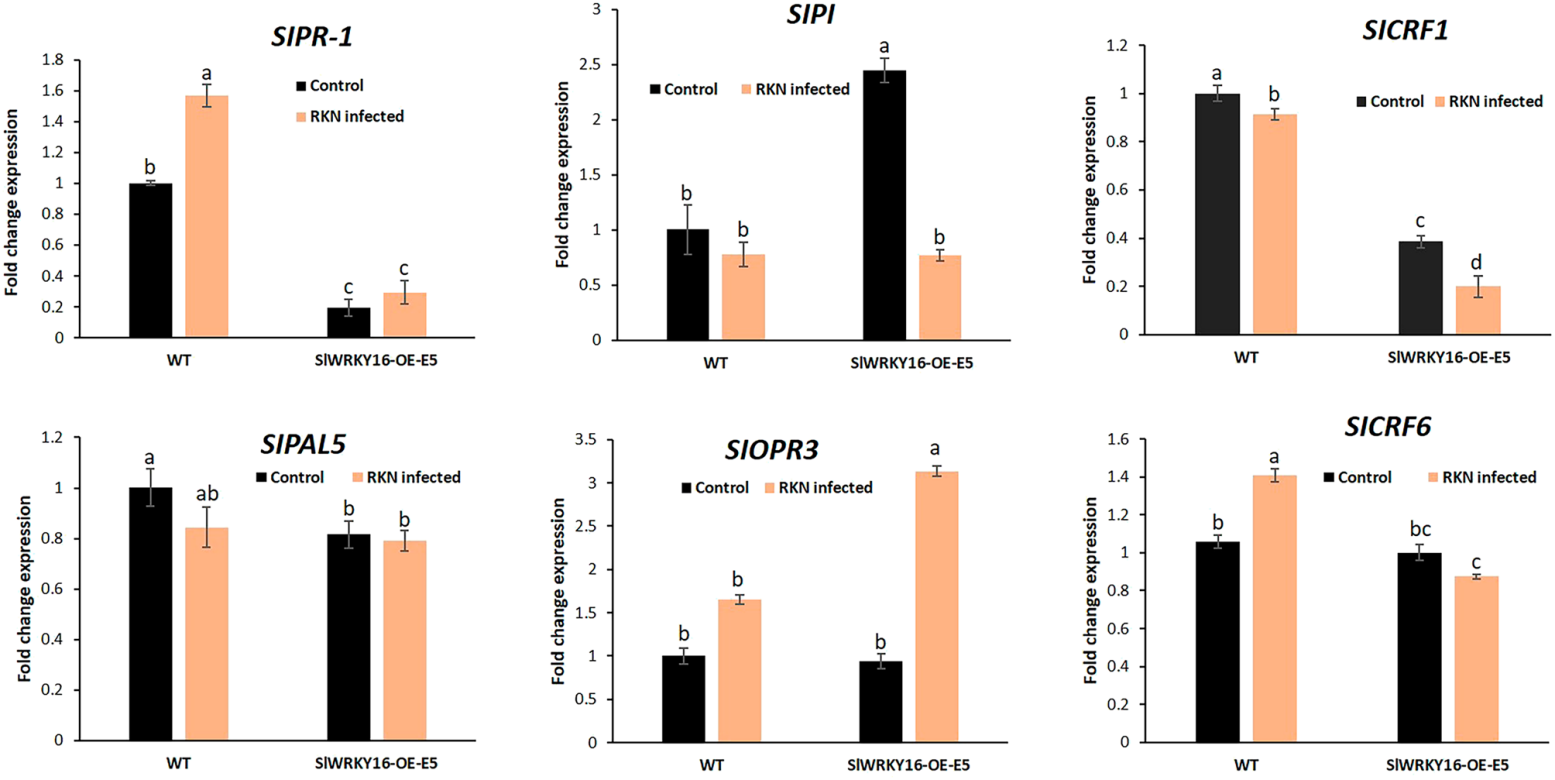
Effect of *WRKY16* overexpression on manipulation of hormonal pathway responses. Expression level of defense-related target genes in roots of *WRKY16*-overexpressing line OE-E5 compared to the control prior to and 1 dpi with *M. javanica*. Total RNA was prepared from infected and noninfected WT control roots and infected and noninfected roots overexpressing *SlWRKY16*. The graph displays the mean and SEM representing the relative transcript levels of these genes in *SlWRKY16*-overexpressing roots (OE-E5) compared to control roots grown under identical conditions. The control expression level was set to zero. All target genes were normalized using the normalization factor, which was calculated as the geometric mean of the expression levels of tomato *β-tubulin*. Each reaction was performed in triplicate, and the results represent the mean of three independent biological replicates. The statistical significance of the differences between OE-E5 and control roots was assessed using the Tukey–Kramer multiple comparison test, and significant differences in expression (P < 0.05) are indicated by different letters above the bars.

**Figure 9:**
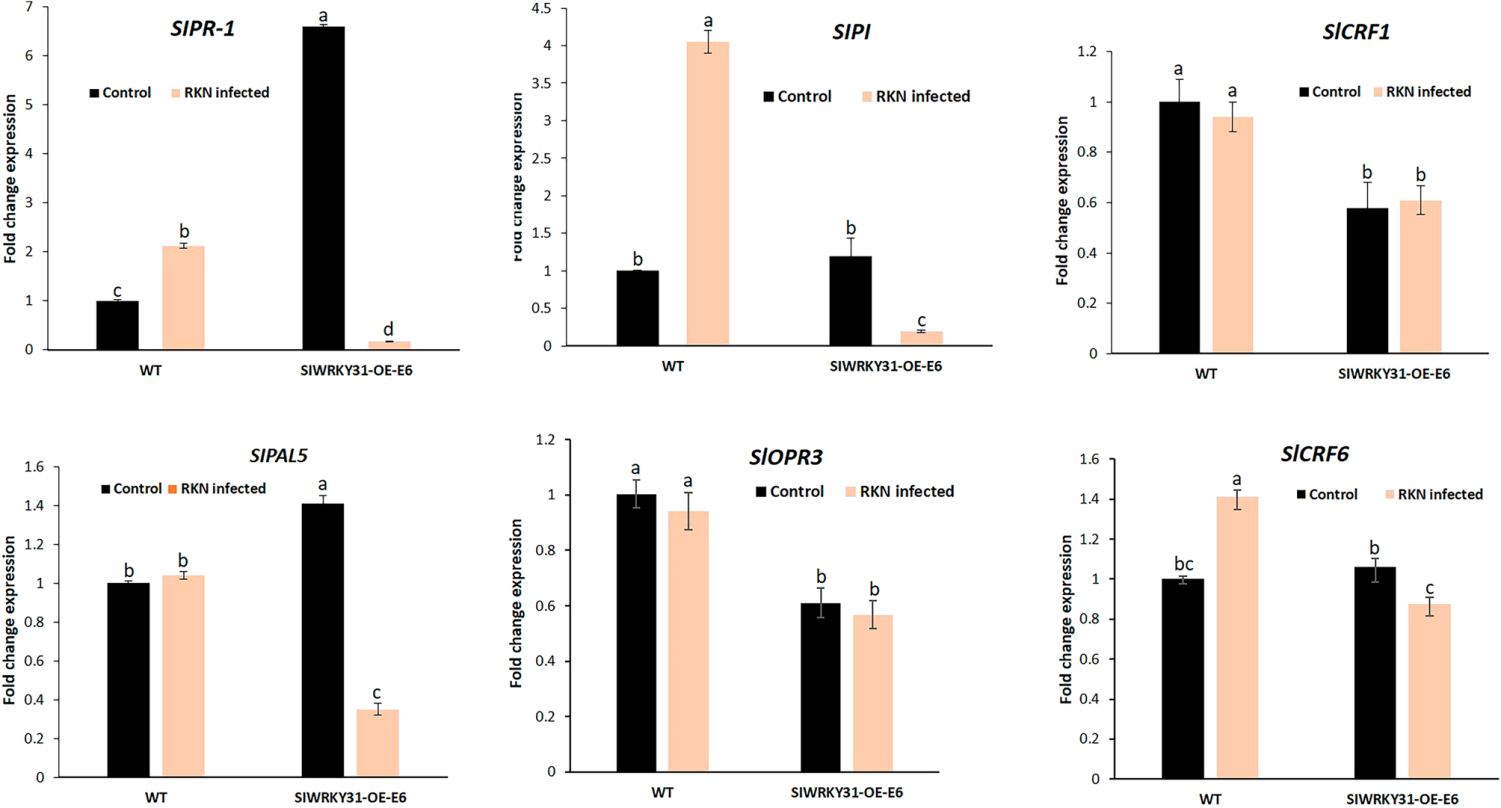
Effect of *WRKY31* overexpression on manipulation of hormonal pathway responses. Expression level of defense-related target genes in roots of *WRKY31*-overexpressing line OE-E6 compared to the control prior to and 1 dpi with *M. javanica*. Total RNA was prepared from infected and noninfected WT control roots and infected and noninfected roots overexpressing *SlWRKY31*. The graph displays the mean and SEM representing the relative transcript levels of these genes in SlWRKY31-overexpressing roots (OE-E6) compared to control roots grown under the same conditions. The control expression level was set to zero. All target genes were normalized using the normalization factor, which was calculated as the geometric mean of the expression levels of tomato *β-tubulin*. Each reaction was performed in triplicate, and the results represent the mean of three independent biological replicates. The statistical significance of the differences between OE-E6 and control roots was determined using the Tukey–Kramer multiple comparison test, and significant differences in expression (P < 0.05) are indicated by different letters above the bars.

To assess the contribution of phytohormone—JA, SA and cytokinin—biosynthesis and regulation to the observed increase in susceptibility of roots overexpressing *WRKY16* (WRKY16-OE-E5) and *WRKY31* (WRKY31-OE-E6), the expression levels of gene markers related to these pathways were investigated using qRT-PCR. The expression of genes commonly used as molecular markers for the activation of the SA signaling pathway was assessed, including pathogenesis-related 1 (PR-1; accession number M69247) and phenylalanine ammonia lyase 5 (PAL5; accession number M90692.1). *WRKY16*-overexpressing lines demonstrated a significant decrease in *SlPR-1* (0.19-fold) compared to wild-type (WT) control roots in both noninoculated (onefold) and inoculated *SlWRKY16*-overexpressing roots (Fig. 8). Expression of the SA-related marker *PAL5* was significantly reduced (0.84-fold) in noninoculated *SlWRK16*-overexpressing root lines compared to the noninoculated WT control roots (onefold) (Fig. 8). This suggested negative regulation of the SA pathway by *SlWRKY16*. Expression of the JA-biosynthesis gene *SlOPR3* (accession number A1486721) significantly increased (3.1-fold) in inoculated *SlWRKY16*-overexpressing roots, whereas the JA marker gene proteinase inhibitor (*PI*; accession number L21194 ) showed a significant increase (2.4-fold) only in the noninoculated *SlWRKY16*-overexpressing line compared to the noninoculated WT control (Fig. 8). Subsequently, we examined the expression levels of cytokinin response factors (CRFs), which are known to be transcriptionally induced by cytokinin and participate in the cytokinin signal-transduction pathway. In the case of SlWRKY16-overexpressing roots, both CRF1 (accession number NM_001247062.2) and CRF6 (accession number XM_004241080.4) exhibited downregulation with fold changes of 0.38 and 0.19, respectively; in response to RKN inoculation compared to the WT inoculated controls. These findings suggest a suppression of the cytokinin signaling pathway in the SlWRKY16-overexpressing line following RKN inoculation (Fig. 8). In the noninoculated *WRKY31*-overexpressing line, both SA-related genes—*SlPR-1* and *SlPAL5*—showed significantly increased expression with fold changes of 6.5- and 1.4-fold, respectively related to the noninoculated control (Fig. 9). Infected WRKY31 overexpressing line showed reduced SIPR-1 gene transcript significantly in compare to WT uninoculated control roots. Regarding JA-related genes, inoculated *WRKY31*-overexpressing lines showed strong repression of *OPR3* (0.56-fold) and *PI* (0.2-fold) compared to control inoculated WT roots.

For the cytokinin-responsive genes, a significant reduction (0.58- to 0.61-fold) in *CRF1* expression was observed in both noninoculated and inoculated *SlWRKY31*-overexpressing root lines, along with a significant reduction (0.87-fold) of *CRF6* in the inoculated *SlWRKY31*-overexpressing line compared to control inoculated WT roots (Fig. 9). Taken together, these results indicate that overexpression of *WRKY16* and* WRKY31* manipulates the signaling pathways of hormones which have been implicated in determining plant immunity during nematode infection.

## Discussion

We explored the involvement of tomato TFs SlWRKY16 and SlWRKY31 during *M. javanica* RKN infection. By utilizing promoter-GUS fusions and qRT-PCR, we demonstrated that the expression of these transcription factors is primarily induced during the first half of the *M. javanica* infection process*,* at the critical time when nematodes are embedded in the colonized roots engaging in feeding sites construction. During the later stage of the infection (28 dpi), the expression of both *SlWRKY1*6 and *SlWRKY31* gradually diminishes, suggesting a plausible function of these transcription factors at the onset of the infection. Accumulating transcriptomic and proteomic data have revealed upregulation of tomato WRKYs upon nematode infection^37–40^. Upregulation of WRKY TFs in response to nematode infection is believed to contribute to the plant's defense against these pathogens, the precise roles of the majority of the 83 WRKYs identified in the tomato genome^14^ in regulating nematode infection remain unclear.

### RKN-induced genes *SlWRKY16* and *SlWRKY31* are members of the IIc subgroup of WRKY TFs

WRKY16 and WRKY31 were classified as members of the IIc subgroup of WRKY TFs, which comprises 16 members in tomato according to Huang et al.^21^. Previous studies have attributed diverse functions to members of this subgroup^59–63^. However, recent research has revealed that WRKY TFs within the same subfamily often share similar functions in regulating the expression of common downstream genes. For instance, in *Arabidopsis*, WRKY8, WRKY28, and WRKY71 from the IIc subgroup redundantly contribute to the acceleration of flowering by directly activating the transcription of FLOWERING LOCUS T and LEAFY^64,65^. Studies have indicated that the group III WRKYs in *Arabidopsis* are involved in different plant defense signaling pathways^66^, whereas our knowledge regarding the role of the relatively large IIc subgroup in disease regulation is limited. An example is AtWRKY12, a member of the IIc subgroup in *Arabidopsis*, which acts in phenylpropanoid pathways by regulating lignin deposition^67^. Mutant plants expressing an atwrky12 mutation, similar to its ortholog in *Medicago truncatula*, exhibit thickened secondary walls in pith cells and a significant increase in stem biomass. AtWRKY12 negatively regulates downstream NAC and CCCH-type zinc finger TFs that control the formation of lignin, xylan, and cellulose^67^. Likewise, suppression of AtWRKY12 orthologs in switchgrass and maize leads to abnormal deposition of secondary cell walls^68^. These findings suggest that the IIc WRKY TF AtWRKY12 has conserved roles across different plant species^69^. Another IIc subgroup WRKY protein, AtWRKY23, participates in root development by controlling auxin distribution^70^. AtWRKY23 was found to be inducible by the plant-parasitic cyst nematode *H. schachtii*^1^, especially during the initial stages of nematode feeding-site establishment, and suppression of AtWRKY23 reduces susceptibility to the parasitic nematode^1^. More recently, group IIc WRKY TFs were identified as key regulators in the response of cotton (*Gossypium hirsutum*) to *Fusarium oxysporum*. Group IIc WRKY TFs directly bind to the W-box element in the GhMKK2 promoter and regulate the transcription of GhMKK2 in response to *F. oxysporum*, thus influencing cotton's response to Fusarium^71^. In conjunction with the findings presented here, it appears that members of the IIc subgroup play a significant role in regulating the plant's response to pathogens, among other functions.

### Both SlWRKY16 and SlWRKY31 negatively regulate root resistance to the RKN *M. javanica*

Since wounding and pathogens both trigger a defense response, transcripts of many genes have been shown to increase after both of these events. It is hypothesized that as nematodes migrate through the root and establish feeding sites, they cause some wounding of the plant tissue^72^. However, several studies have shown that many transcripts induced by both processes might have a different role within host cells, as shown for extensin transcripts induced following wounding as compared to RKN infection. Wounding experiments on WRKY16:GUS tomato roots enabled exploring wounding-mediated activation of WRKY TFs^20^ as part of the general wound-response mechanism intended to enhance tolerance to wounding. Previous studies have indicated that JA biosynthesis contributes, partially to substantially, to the upregulation of most *WRKY* genes upon wounding^73,74^. However, their suppression by SA is also observed, because JA and SA have largely antagonistic functions^75–77^.

While wound-induced suppression of *SlWRKY16* expression was observed, a slight increase in *SlWRKY31* expression was also noted following the wound, although it was not significant. Paradoxical to the expected function of WRKY proteins in plant defense^20,78^, throughout our study, both *WRKY16*-and *WRKY31*-overexpressing tomato roots showed increased susceptibility to *M. javanica* infection.

This paradoxical observation could be attributed to two potential scenarios. Firstly, it is possible that both WRKY proteins function as negative regulators of basal defense. Negative regulators are known to modulate the plant's response to nematode infection by suppressing or attenuating the plant's defense mechanisms. It is important for plants to regulate their response to nematode infection, as an overactive defense response can lead to excessive cell death and tissue damage, while an insufficient defense response can result in increased susceptibility to infection. Thus, one option is that upon RKN infection, during migration and feeding-site establishment, WRKY16 and WRKY31 operate as negative regulators of plant defense responses. This hypothesis might also be supported by the expression profiling of marker genes responsive to the defense-associated phytohormones SA, JA and cytokinin, indicating that in infected *SlWRKY16*-overexpressing lines, decreased expression of *PR-1*, *CRF1* and *CRF6*, along with induced expression of *OPR3*, is observed. Similarly, *SlWRKY31*-overexpressing lines show suppressed expression of the SA signaling marker genes *PR-1* and *PAL5*, and the JA-related genes *PI* and *OPR3*, as well as the cytokinin-responsive gene *CRF1* following inoculation. These results suggest that overexpression of *SlWRKY16* and *SlWRKY31* leads to manipulation of the plant's phytohormone regulation upon RKN inoculation.

Enhanced disease resistance in plants is often associated with the upregulation of *PR-1* gene transcripts, which play a crucial role in the SA-mediated defense pathway. In our study, we investigated the impact of *WRKY16* and *WRKY31* genes on *PR-1* gene expression in transgenic tomato roots susceptible to nematode infection. Our findings demonstrated a significant decrease in SlPR1 marker transcript levels in susceptible transgenic roots overexpressing *WRKY16* and *WRKY31*. This suggests that WRKY16 and WRKY31 act as negative regulators of pathogen-induced PR gene expression. These results align with a previous study conducted by Xing et al.^28^, which also reported similar results in *WRKY48 *overexpressing plants. In their study, the overexpression of WRKY48 resulted in enhanced susceptibility to *P. syringae* and a concomitant reduction in the expression of the SA-regulated PR1 gene. Importantly, the reduction in *PR-1* gene expression did not compromise the accumulation of SA in the plants, highlighting the specificity of the regulatory mechanism. Moreover, another report also indicated a reduction in PR-1 transcript levels in plants overexpressing *WRKY7*^79^, rendering them susceptible to *P. syringae*. Similarly, Yokotani et al. demonstrated that the overexpression of *OsWRKY76* in rice plants resulted in a significant increase in susceptibility to *M. oryzae* fungi^80^. To gain insights into the underlying mechanisms, microarray analysis was employed. The analysis provided valuable insights, indicating that the overexpression of OsWRKY76 suppresses the induction of *PR1*, *PR10b*, and *PR15* genes following inoculation with the blast fungus^80^. Overall, our study and the aforementioned studies shed light on the regulatory role of WRKY genes, such as *WRKY16*, *WRKY31*, *WRKY48*, *WRKY7*^79^, and *OsWRKY76*, in the modulation of PR gene expression and plant susceptibility to various pathogen.

The expression of PAL (Phenylalanine Ammonia-Lyase) plays a positive role in the activation of SA signaling pathways. This activation leads to the production of antimicrobial secondary metabolites that protect the plant from pathogens. In our study, we observed that SIPAL5, a marker for SA signaling, was significantly downregulated in WRKY16 and WRKY31 overexpressing lines that showed susceptibility to nematode. Similar findings were reported in CaWRKY70 overexpressing chickpea shoots, where the transcripts of CaPAL were downregulated following *Fusarium oxysporum* infection^81^. Furthermore, a recent study demonstrated that the overexpression of *SlWRKY46* in tomato plants increased their susceptibility to *Botrytis cinerea*, and was associated with a reduction in PAL^82^. This suggests that the overexpression of WRKY16 and WRKY31 negatively affects PAL expression and, subsequently, the JA signaling pathway.

Protease Inhibitor (PI) is a key gene in the jasmonic acid (JA) pathway. In our study, we found that *WRKY31*-overexpression roots displayed increased susceptibility to nematodes, which was accompanied by a reduction in *PI* gene transcript levels. Similarly, as previously reported, over-expression of *SlWRKY46* resulted in a lower level of *PI* and a more severe phenotype after *Botrytis cinerea* infection compared to wild-type plants^82^. Our results are consistent with the findings of the aforementioned studies and confirm that WRKY31 may negatively regulate the resistance of tomato roots to nematodes by affecting the jasmonic acid pathway. Regards alteration in other hormone regulating marker genes *OPR3*, *CRF1*, *CRF6*, no indication for WRKYs implication was indicated so far, however their differential expression in overexpressing lines might support their regulation by the respective SlWRKY.

Several other WRKY genes have been identified with a similar function as negative regulators of basal defense responses in plants. Notably, mutations in Arabidopsis WRKY7, WRKY8, WRKY11, and WRKY17 have been shown to enhance basal resistance to virulent strains of the bacterial pathogen *P. syringae*^79,83–85^. The *Arabidopsis* homologs AtWRKY71, AtWRKY48, and AtWRKY23 negatively regulate the plant's immunity to *P. syringae* and cyst nematodes^28^. In recent study, it has been demonstrated that WRKY48, along with two closely related proteins, WRKY38 and WRKY62, act in an additive manner as negative regulators of basal defense against *P. syringae*^27,28^. Interestingly, our phylogenetic analysis indicated high similarity of SlWRKY16 to AtWRKY48 (40.62%) and of SlWRKY31 to AtWRKY48 (43.06%), emphasizing their likelihood of also functioning as negative regulators of basal defenses.

The notion that WRKY16 and WRKY31 are subject to hormonal regulation is further supported by the promoter motif analysis, which indicated the presence of a *cis*-acting element involved in SA responsiveness (CCATCTTTTT), a W-box recognized specifically by SA-induced WRKY DNA-binding protein (TTGACC), and a pathogenesis-related element (TGTCA) in both promoter sequences. Similarly, decreased expression of cytokinin-related genes *CRF1* and *CRF6* could be explained by gibberellin hormone regulatory elements found on WRKY16 and WRKY31 as a mutually antagonistic interaction between gibberellin and cytokinin in tomato has been suggested^86^.

Several other WRKY genes have been identified with a similar function as negative regulators of basal defense responses in plants. Notably, mutations in Arabidopsis WRKY7, WRKY8, WRKY11, and WRKY17 have been shown to enhance basal resistance to virulent strains of the bacterial pathogen *P. syringae*^79,83–85^. The *Arabidopsis* homologs AtWRKY71, AtWRKY48, and AtWRKY23 negatively regulate the plant's immunity to *P. syringae* and cyst nematodes^28^. In recent study, it has been demonstrated that WRKY48, along with two closely related proteins, WRKY38 and WRKY62, act in an additive manner as negative regulators of basal defense against *P. syringae*^27,28^. Interestingly, our phylogenetic analysis indicated high similarity of SlWRKY16 to AtWRKY48 (40.62%) and of SlWRKY31 to AtWRKY48 (43.06%), emphasizing their likelihood of also functioning as negative regulators of basal defenses.

The second scenario that might explain the enhanced susceptibility of *SlWRKY16* and *SlWRKY31* lines to *M. javanica* infection is hijacking of both WRKYs by the invading nematode. It has been suggested that plant-parasitic nematodes exploit the developmental processes of their host plants to establish their nematode feeding sites (NFS)^87^. These nematodes manipulate the expression of plant genes for their own advantage. Given that both WRKY16 and WRKY31 play a role in regulating hormone-related genes, it might be that their expression supports the hormonal conditions that underlie feeding-site expansion and nematode development. Considering the dependence of plant-parasitic nematodes on nutrient acquisition from their host plants, it is tempting to speculate that the SA signaling pathway in the hosts would undergo constant activation.

The results here, where overexpression of both *SlWRKY16* and *SlWRKY31* suppressed SA marker genes and resulted in increased susceptibility is in agreement with those of Wubben et al.^88^ who showed enhanced susceptibility in SA-deficient mutants infected with *H. schachtii*. This suggests a possible explanation that the nematode can evade plant defense signals by activating both WRKYs, thereby creating a hormone environment conducive to nematode development within the roots.

In conclusion, WRKY TFs play a crucial role in the intricate regulation and precise coordination of signaling and transcriptional networks that govern plant responses to wounding and RKN infection. Our results place *SlWRKY16* and* SlWRKY31* as negative regulators of plant defense, because overexpression of both TFs affected the expression of defense-related genes, enhancing susceptibility to nematode infection. Further investigation into the regulatory mechanisms underlying *SlWRKY16* and *SlWRKY31* overexpression may uncover a pivotal point of convergence in the regulatory pathways involved in the plant's responses to both wounding and pathogen infection. These findings highlight the importance of WRKYs in regulating RKN-induced responses.

### Supplementary Information

Supplementary Figure S1.

Supplementary Figure S2.

Supplementary Figure S3.

Supplementary Tables.

Supplementary Legends.

## Data Availability

All of the data generated or analyzed has been provided in the manuscript and supplementary data. The accession IDs links to the sequence(s) are available below and throughout the text in the manuscript Solyc07g056280.2.1; Solyc05g053380.2.1; Solyc02g071130.2; Solyc12g011200.1; Solyc01g058540.2.1; Solyc01g079260.2.1; AT1G29860.1; AT4G18170.1; AT1G69310.1; AT5G49520.1; AT5G49520.1; AT2G47260.1; LOC_Os01g47560.1; LOC_Os03g55080.1; LOC_Os05g50610.2; LOC_Os05g49100.1; U60482.1; NM_001247878.1; M69247; M90692.1; L21194; A1486721; NM_001247062.2; XM_004241080.4.
