# Supplementary figures and images for: SlWRKY16 and SlWRKY31 of tomato, negative regulators of plant defense, involved in susceptibility activation following root-knot nematode Meloidogyne javanica infection
Source: Sci Rep. 2023 Sep 5;13:14592. doi: 10.1038/s41598-023-40557-z (PMC10480479; doi:10.1038/s41598-023-40557-z)

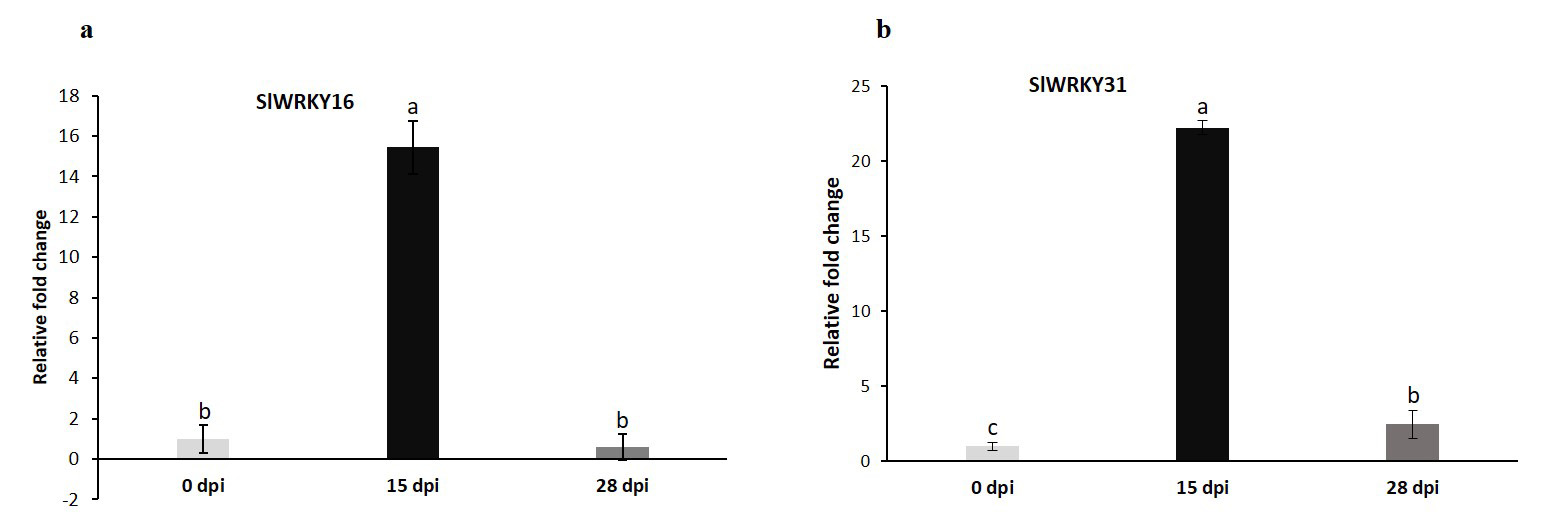

Supplement: Supplementary file 1 — Supplementary Figure S1. [file 41598_2023_40557_MOESM1_ESM.jpg]

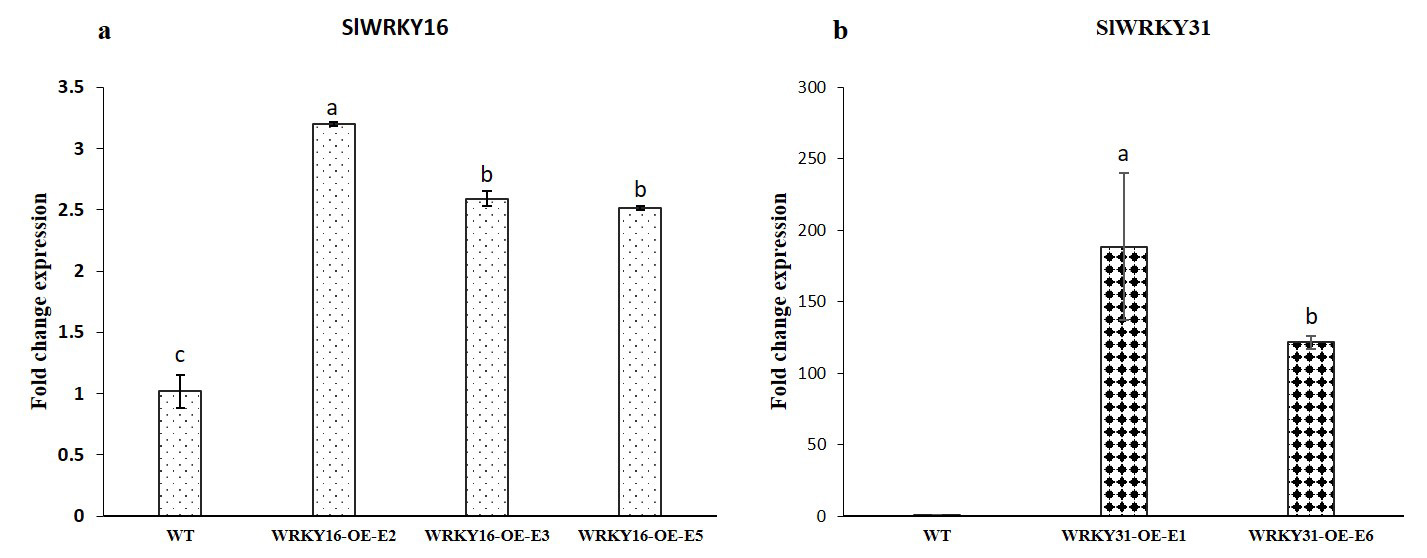

Supplement: Supplementary file 2 — Supplementary Figure S2. [file 41598_2023_40557_MOESM2_ESM.jpg]

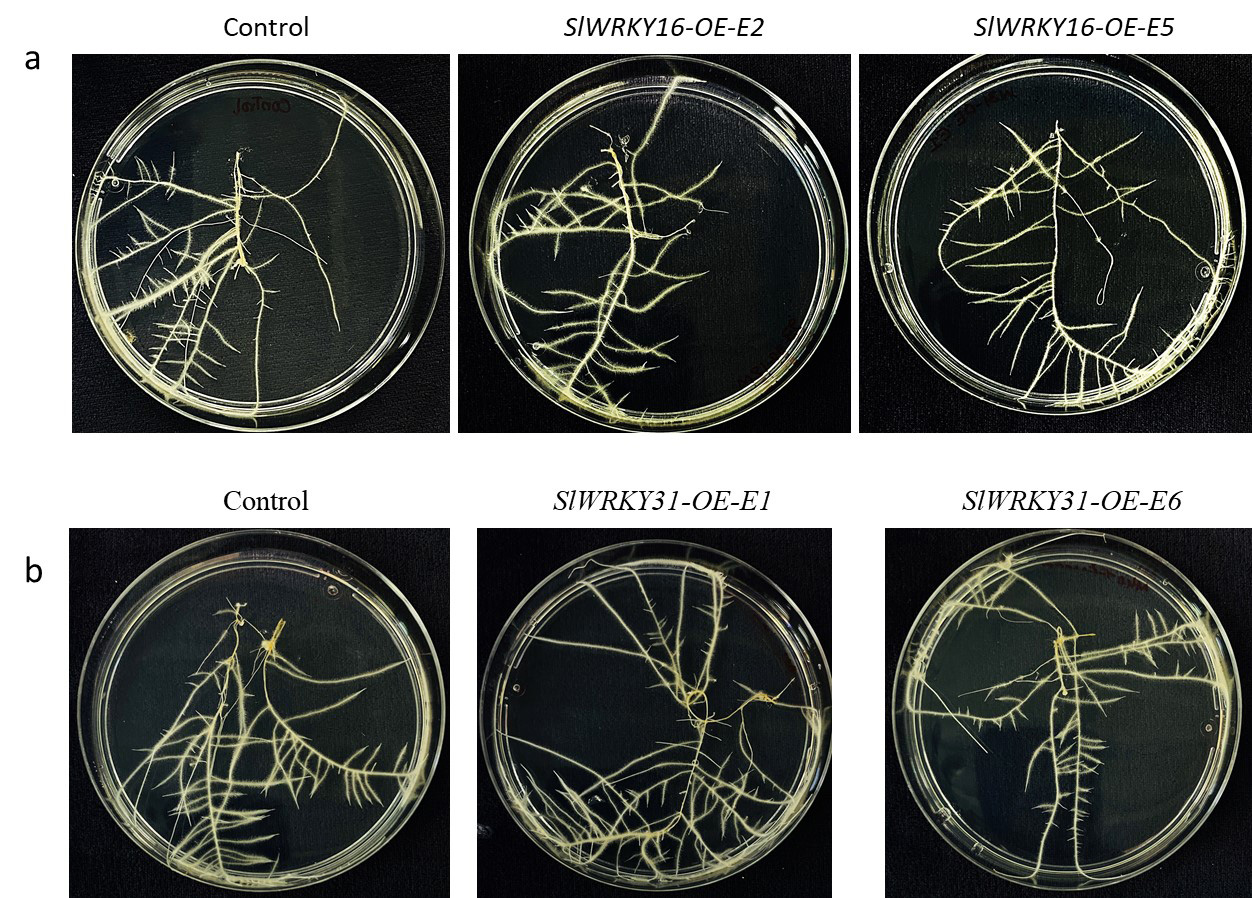

Supplement: Supplementary file 3 — Supplementary Figure S3. [file 41598_2023_40557_MOESM3_ESM.jpg]
