# Supplementary material for: SlWRKY16 and SlWRKY31 of tomato, negative regulators of plant defense, involved in susceptibility activation following root-knot nematode Meloidogyne javanica infection
Source: Sci Rep. 2023 Sep 5;13:14592. doi: 10.1038/s41598-023-40557-z (PMC10480479; doi:10.1038/s41598-023-40557-z)
Supplement: Supplementary file 4 — Supplementary Tables. [file 41598_2023_40557_MOESM4_ESM.docx]

**Table legends**

**Supplementary Table S1.** Primers used for gene expression, cloning and promoter analysis of *WRKY16* and *WRKY31*.

**Supplementary Table S2.** Real-time RT-PCR primers used for *SlWRKY16* and *SlWRKY31* defense-related genes.

**Table S1.**

| **Gene name** | **Primer 5'→ 3'** | **Amplicon size (bp)** |
| --- | --- | --- |
| Primers used for overexpression | | |
| *SlWRKY16* | F-ATGTCTGATAATAACCCTTTTAATCATGATTA  TG | 968 |
|  | R-GTACTTCACTAAATGCTCATGGCTCT |  |
| *SlWRKY31* | F-ATGGAGAAAGAAAATAATGTAAGAACAG | 888 |
|  | R-CTACTCTTCTATAAGATCACTTCTCATAC |  |
| Primers used for promoter analysis | | |
| *SlWRKY16* | F-GAAATTCTCGTGACCTAATCCC | 1436 |
|  | R-GTAGAGAAGCAAAAAAAGGGGG |  |
| *SlWRKY31* | F-ATGGCCCATGACAATGATGAG | 1494 |
|  | R-CGAAAGAGATAATGTAGAGAATTTGTCTAAG |  |
| qRT PCR primers | | |
| *SlWRKY16* | F-GCCAATAACACACAAAACCCTC | 134 |
|  | R-ATCCCAAAAGCACTTGACAAAG |  |
| *SlWRKY31* | F-TTCCAAGGAGCTACTAT | 124 |
|  | R-CATGGATGTGTGTGAAC |  |
| Gene confirmation primers | |  |
| GFP | F-TGGTGCCCATCCTGGTCGAGC | 341 |
|  | R-GCTCGATGCGGTTCACCAGGG |  |
| GUS | F-TGATAGCGCGTGACAAAAA | 92 |
|  | R-CGAAATATTCCCGTGCACTT |  |

**Table S2.**

| **Primer name** | **Primer 5'→ 3'** |
| --- | --- |
| *SlCRF1* | F-AAA TCG ATC GGC GAC AGA AAA CG |
|  | R-CCC GAA TCT CTG CAG CCC AA |
| *SlCRF6* | F-GGC GTT GAA AGG GAA GGA AGT |
|  | R-TAA CCA GAG CCT AAC GCG ACG |
| *Sl- β-actin* | F-ATGTATGTTGCCATCCAGGCT |
|  | R-TGTGGCTGACACGATCTCCA |
| *Sl-β-tubulin* | F-ACCATTTGATCTCTGCAACCATG |
|  | R-TTCACAGCCAATTTCCTCAGG |
| *SlPR1a* | F-GAGGGCAGCCGTGCAA |
|  | R-CACATTTTTCCACCAACACATTG |
| *SlPAL5* | F-CAATGGCTTCTTACTGCTCGG |
|  | R-CATCTTGGTTGTGTTGCTCAGC |
| *SlOPR3* | F-TTGGCTTAGCAGTTGTTGAAAG |
|  | R-TACGTATCGTGGCTGTGTATCA |
| *SlPI* | F-TTGCTCTCCTCCTTTTATTTGG |
|  | R-GCAAGCCTTGGCATGTTC |

s
