# Supplementary material for: SlWRKY16 and SlWRKY31 of tomato, negative regulators of plant defense, involved in susceptibility activation following root-knot nematode Meloidogyne javanica infection
Source: Sci Rep. 2023 Sep 5;13:14592. doi: 10.1038/s41598-023-40557-z (PMC10480479; doi:10.1038/s41598-023-40557-z)
Supplement: Supplementary file 5 — Supplementary Legends. [file 41598_2023_40557_MOESM5_ESM.docx]

**Supplementary Figures legends:**

**Supplementary Figure S1.** Expression of tomato *WRKY* genes in response to *M. javanica* infection. Expression of *SlWRKY16* (a) and *SlWRKY31* (b) in tomato roots was determined by qRT-PCR at 0, 15 and 28 days postinfection (dpi) and in uninfected root segments (control; 0 dpi). Data include three independent biological and three technical replicates. *WRKY16* and *WRKY31* expression values are relative to uninfected control roots and were normalized using tomato *β-tubulin* as a reference gene. Bar shows SEM. Different letters above the bars indicate significant differences by Tukey’s HSD test (*P* < 0.05).

**Supplementary Figure S2.** Transcript levels of transgenic *WRKY*-overexpressing lines of tomato by qRT-PCR. (a) *SlWRKY16* and (b) *SlWRKY31* expression in transgenic lines vs. WT control. Tomato *β-tubulin* housekeeping gene was used for normalization.

**Supplementary Figure S3:**  Phenotypic comparison of tomato SIWRKY16 and SIWRKY31 transgenic overexpressing roots with control. a) SlWRKY16 transgenic lines; WRKY16-OE-E2 and WRKY16-OE-E5 and control empty vector b) SIWRKY31 roots: WRKY31-OE-E1 and WRKY31-OE-E6 and control empty vector.
